# Investigation of the Cultural Context of Sugars Consumption Behavior in Low-Income Mexican-American Women

**Published:** 2017

**Authors:** Tanya J. Benitez, Colleen Keller, Kathryn Coe, Natasha Tasevska

**Affiliations:** Arizona State University, Phoenix, AZ; Arizona State University, Phoenix, AZ; Indiana University – Purdue University, Indianapolis, IN; Arizona State University, Phoenix, AZ

**Keywords:** Sugars, diet, Mexican-American women, culture, focus groups

## Abstract

Despite the recent federal dietary recommendations to limit consumption of added sugars to less than ten percent of daily caloric intake, there is a gap in published literature examining the influence of food preparation behaviors on sugars consumption among low-income Mexican-American women. The purpose of the study was to describe the cultural context of Mexican-American women in procuring, preparing and presenting added sugars in their families’ diets.

Five focus groups were conducted to examine sugars consumption behavior in thirteen overweight/obese low-income Mexican-American women ages 27–40 years. Themes that emerged during the sessions included: changes in food procurement to include high-sugar foods and sugar-sweetened beverages following migration from Mexico to the U.S.; children’s influence on what was bought and consumed in the household; changes in household diet when relatives prepared food; and influence of family traditions/extensive social gatherings and traditional foods during holidays on sugars consumption. Culturally relevant factors influencing sugars consumption were identified. We report strategies that can be used in public health interventions to reduce sugars intake among low-income Mexican-American women; such strategies must acknowledge cultural and contextual factors of social ties, the role of family members in influencing diet, and importance of maintaining traditional foods and cultural celebrations.

## INTRODUCTION

There has been accumulating evidence to show that high sugars consumption increases the risk of overweight and obesity (Malik, Schulze, & Hu, 2006; Te Morenga, Mallard, & Mann, 2013), a major risk factor for cardiovascular disease (CVD) and type 2 diabetes. Furthermore, a recent systematic review and meta-analysis found that independent of sugars’ effect on body weight, their intake was associated with raised blood pressure and blood lipids (Te Morenga, Howatson, Jones, & Mann, 2014). Mexican-American females have disproportionately higher rates of obesity (75%) and metabolic syndrome (41%) than non-Hispanic white women (58% & 32%, respectively) (Ervin, 2009), and have experienced rapid increases in the prevalence of obesity (BMI ≥30) (Flegal, Carroll, Kit, & Ogden, 2012) and metabolic disease in recent years. High insulin resistance and poor glycemic control found in Hispanics (Goran, Bergman, Cruz, & Watanabe, 2002; Haffner et al., 1996) suggest that consumption of sugars may be an important dietary determinant of obesity and metabolic disease risk in this rapidly growing ethnic group; thus, making this exposure a critical candidate for dietary interventions.

In efforts to reduce lifestyle-related chronic diseases in the U.S., recent modifications to the federal dietary guidelines recommend limiting consumption of added sugars to less than ten percent of daily caloric intake (U.S. Department of Health & Human Services & U.S. Department of Agriculture, 2015). Intervening to reduce added sugars consumption requires an understanding of contextual nuances among Mexican-American women, such as migration to the U.S., which can shape dietary behavior. A substantial body of knowledge exists on dietary changes among Mexican-American immigrants following the transition to the U.S, including limited time for food preparation, greater reliance on fast-foods and snacks, increased consumption of processed foods, meat, sweets and sugar sweetened beverages (SSB), and decreased consumption of fruits, vegetables, legumes, beans and grains. These changes likely stem from limited access to quality food, local abundance of fast food outlets, social isolation, and changes in food preparation and eating rituals (Ayala, Baquero, & Klinger, 2008; Duffey, Gordon-Larsen, Ayala, & Popkin, 2008; Guarnaccia, Vivar, Bellows, & Alcaraz, 2012; Himmelgreen, Daza, Cooper, & Martinez, 2007). Furthermore, Hispanics in the U.S. tend to have lower income than non-Hispanic whites (DeNavas-Walt & Proctor, 2015), which has been associated with higher intake of SSB and fruit drinks (Ervin & Ogden, 2013; Han & Powell, 2013; Thompson et al., 2009). It has been recently reported that factors such as culture and tradition, family, and attitudes towards healthy eating, play an important role in influencing food preparation behaviors among Mexican-Americans (Smith, Dunton, Pinard, & Yaroch, 2016). Such findings are essential for informing the development of dietary interventions, as Mexican-American mothers’ food preparation behaviors at home can influence the dietary habits of the entire family. Yet, little is known about culture-related and tradition-related food preparation behaviors that influence added sugars among Mexican-American women.

While interventions aimed at reducing the consumption of added sugars may be particularly effective in Mexican-American women, further understanding of the cultural context that shapes added sugars consumption behavior in this population is necessary to inform the development of such an intervention. To address this gap in published literature, we conducted a focus group study in Mexican-American women. The purpose of our study was to explore the added sugars consumption behavior in young Mexican-American mothers, as it relates to their culture, tradition, and eating habits. We describe the cultural context of Mexican-American women of childbearing age in procuring, preparing and presenting added sugars in their families’ diets.

## METHODS

### Participants and Study Design

Thirteen low-income Mexican-American women age 27–40 years residing in central Phoenix with a BMI of 25–40 kg/m^2^ were recruited from the participant pool of “Madres para la Salud” (thereafter referred as *Madres*). The study participants had provided consent to be re-contacted for future studies. Madres was a 12-month randomized control trial involving 139 postpartum sedentary low-income Hispanic women, completed in 2010, which investigated the effect of a culturally-tailored social-support-based physical activity intervention on changes in body weight, body fat, and metabolic risk factors (Keller et al., 2014). Five focus groups with a total of 13 participants were held between January and May 2015. Inclusion criteria included 1) participating, having a friend participate, or serving as *promotora* in *Madres* (Keller et al., 2014), 2) age between 23–40 years, and 3) a BMI between 25 and 40 kg/m^2^. Each study visit was approximately 2.5 hours long, and comprised of obtaining an informed consent, completing an interviewer-administered demographics questionnaire, and participating in a 1.5 hour focus group session. A bilingual bicultural moderator trained in qualitative interviewing collected data and led the focus group sessions. All study materials were translated and back-translated from English to Spanish by bilingual members of the research team to ensure accuracy and comprehension of the questions. The focus group study was approved by the Arizona State University Institutional Review Board (IRB).

### Data Collection

#### Demographics questionnaire

Following the consent process, participants were administered a demographics questionnaire, which enquired about their age, marital status, education level, work status, household size, preferred language, and years lived in the U.S.

#### Focus group script

The focus group script was developed after reviewing the literature for knowledge gaps on sugars consumption in Mexican-American women. The script included topics on food procurement, preparation and presentation, and family traditions in the Hispanic culture as they related to sugars consumption (Table 1; for focus group training guide see Appendix). We were particularly interested in acquiring information on the contribution of traditional foods to their sugars intake, and the impact of holidays and the effect of family on sugars intake. The focus groups were conducted in Spanish and English, according to participant preference; there were four Spanish and one English language focus group. All focus group sessions were audio recorded, and once transcribed, they were translated into English. Translated transcripts and audiotaped interviews were then compared to ensure accurate translation.

### Data Analysis

Qualitative content analysis guided description of the data (Hsieh & Shannon, 2005), with the identification of data codes and data categories, and included an iterative approach and constant comparison in data analysis. Qualitative content analysis was used to capture and extend knowledge and understanding of cultural and contextual sources for added sugars intake among Mexican-American women. Data analysis took place at two levels. At the first level, we reviewed the statements from the focus groups to understand the data in context. At the second level, data were coded across transcripts using the constant comparison method, to group related concepts into categories. The authors read the transcripts independently, and developed initial coding schemes. Identified categories were shared and discussed by team members. Data were then classified to formulate distinct categories and synthesize themes, and discussion and analysis continued until agreement on the categories was reached (Hsieh & Shannon, 2005). Following the development of contextual categories, the data were organized into the broader categories of food procurement, preparation and presentation and related to the norms and values of Mexican-American women.

## RESULTS

Participants (N=13) were Mexican/Mexican-American females ages 27–40 years (mean=35.3, SD= 4.0) who were either overweight (n=6) or obese (n=7) with a mean BMI of 29.7 kg/m^2^ (SD=3.1) (Table 2). All participants reported annual household income of less than $50,000, and five less than $20,000. Only one participant was born in the U.S., while the others had migrated from Mexico and reported living in the U.S. between 8–27 years (median=15 years). All 13 participants did the majority of household food preparation, and 12 out of 13 were responsible for household food shopping. Participants’ demographics characteristics have been reported in Table 2.

The identified themes from the focus group sessions were grouped into three broader categories: 1) food procurement, 2) food preparation, and 3) food presentation.

### Food Procurement

Changes due to migrating from Mexico to the U.S. was a commonly recurring theme during the focus group sessions. Prior to coming to the U.S., participants valued purchasing fresh fruits and vegetables, a behavior learned from their parents or grandparents in Mexico. The majority reported that despite earning less in Mexico, their families were able to afford fresh fruits, whereas the sugary foods or SSB were cost prohibitive. Once they arrived in the U.S., they reported buying more SSB or sweets as these items became far more accessible and convenient to consume. One participant highlighted this issue when she said:

“Well, yes, I have noticed that here it is easier to get sweets. I do not know if it’s because we make more than in Mexico… because in Mexico, one brings fresh things, the fresh food and eats purely healthy…well, here, it is easier (to eat sugars), because you go to the store, and there is a sale… four boxes of soda for ten and, you say, well, ten is a bunch of soda. Here, yes, there is more (sugar) and the difference from here and there (Mexico) is that the food was healthier.”

Additionally, participants reported that the frequency of their shopping trips changed from Mexico to the U.S. In Mexico, participants tended to go to the store several times a week, always purchasing fresh foods, while in the U.S. they reported going once a week and would therefore adjust their purchases to include less fresh foods and more calorie dense processed foods typically high in added sugars. As one participant expressed:

“When I was little, I lived with my Grandma in Mexico. So, the food that she made was always fresh, traditional, it was every day that we went to the grocery, the butcher. Then we bought what we would be eating that day…. Then, when I started living with my parents (in the U.S.), I was little older, and everything changed completely, because they worked all of the time. Then, they had lots of soda, something that I did not drink when I was younger.”

Another category that emerged was the children’s influence on what was bought and consumed in the household. Several participants reported that when their children went shopping with them, foods high in sugars, such as SSB, sweets and candies, were purchased more often. One participant stated that *“I try not to take my kids because when I do, they want to get stuff that they know we are not supposed to get.”* While others agreed that their children often want the foods that are unhealthy, they often gave into their children’s demands by purchasing high sugar foods in order to please them. The items described were mostly high sugar beverages like SSB, fruit drinks and Kool-Aid. Another participant explained that she often purchases unhealthy foods and snacks because: “*in my house, if there are no cookies or little pancakes and all those little baggies, there is no food according to my kids*.”

While participants demonstrated the ability to make a connection between nutrition/sugars intake and disease risks, food procurement was influenced by participants’ low nutrition knowledge and misinformation. Throughout the sessions, for example, one participant did not understand what processed foods meant or how such foods would impact her health. Another participant described thinking that the food label values referred to the entire package rather than one serving only. Other participants did not understand the difference between starches and sugars- “*we eat baked potatoes, but there then there you go again with your sugar intake*.” Moreover, participants did not understand the difference between types of sugars and misperceived some as healthier, such as brown sugar.

### Food Preparation

A major theme in the food preparation category was the effect of others on sugars consumption patterns. Participants spoke about changes in their usual household dietary consumption pattern that occurred when other family members (primarily spouses, in-laws and children) prepared or purchased the food. These changes often resulted in increased consumption of SSB, convenience foods or traditional meals high in added sugars. For example, one participant discussed how her husband tended to use large amounts of sugar in his cooking in order to please the children. Another participant said: *“My husband likes to cook and my children like my husband very much because my husband makes all for them… he puts marmalade and cream in the pancakes.”*

Extended visits from in-laws were cited as being influential on participants’ dietary patterns. One participant stated that “*ever since she (her mother-in-law) came, she’s been an influence on whatever we’re eating…because we just eat whatever she’s making*.” Another participant spoke about her mother-in-law’s six-month visit; during this time, her mother-in-law took over the household cooking, and thus her shopping and everyone’s diet in the household had been modified to include more sugars. She noted that her mother-in-law would tell her that they’re almost out of sugar shortly after participant purchased two four-pound bags. She stated that saying:

“I think it’s because she uses a lot of sugar in our drinks or some cooking. So that’s what she adds more sugars in, and then, the warm drinks, she’s a big coffee drinker too, so, I noticed that I went up on drinking coffee, too.”

Similarly, other participants spoke about how observing others consume high sugar foods influenced their own consumption of added sugars. For example, another participant stated that when her mother-in-law came to visit, she found herself adding sugar to coffee despite not usually drinking it with sugar. Participants spoke how their children were acquiring habits based on their own behavior and actions and, therefore, they were being “*motivated by our children…to start eating right*.”

Children’s influence on food preparation. Participants highlighted that their children spend more time at school than they do at home, which made them more accustomed to highly processed foods than the traditional Hispanic dishes and often wanted foods such as pizza, hamburgers and cookies. They reported that their children often bake desserts and cookies at home, based on recipes that they find online, hence creating an atmosphere where high-sugars foods are readily available at home. This was described by a participant when she said:

“Oh, I have a ten year old daughter. She makes anything you tell her to make, like … She searches for it on the Internet, then she buys what she needs, and makes it. Although we try to gauge what they eat, but like I tell you, well, she does it two or three times per week. She makes a cake, cookies… then she makes it for everyone. She even puts it there, right in front in a serving dish. So that one can see them! So that you eat all of them! Then we tell her how good they are and she makes more.”

Family traditions and behaviors learned from generation to generation were reported as an important determinant of participants’ sugars intake. Most participants reported learning how to prepare traditional foods and drinks from their mother, older sister(s), mother-in-law, or grandmothers. Several participants described how they learned making drinks such as *aguas frescas* [water flavored with fresh fruits and sugar] from their mothers, which they commonly consumed in Mexico. When one participant spoke about this, she said:

“I also learned with my mother, at home, that is how I learned to make the beverages… we put sugar to one’s taste; my mother also use to do to her own taste, and they were not (as) sugary.”

“… in the house [at home], the drinks are made from strawberries, watermelon, melon, and yes, I put some sugar, but I do not put sugar when I do the orange drink.”

“Well look over there in Mexico, normally, one does not have money to buy craving, you want to go buy a pack of cookies or drink soda every day, in reality, you do not have [money] for those things. So over there for example agua fresca is made…or you drink water. It is more like that you don’t really have to buy juzgeras [cravings]. Here, I have enough to purchase the cookies, the bread, the soda and all of that.

### Food Presentation

The environment in which food was consumed also influenced the amount of food and sugars consumed. The Mexican-American women in this study reported that traditions or habitual behaviors were a source of sugars in food presentation. They described traditional Mexican foods, such as *Piloncillo* [cone-shaped candy made of unrefined whole cane sugar] and *aguas frescas* as major sources of sugars in their diet. Several participants reported that they only consumed SSB with certain types of meals. Multiple participants described only drinking water throughout the day but when they ate a certain meal, they consumed sodas. One participant noted that when she ate a meal at a restaurant, she consumed three cans of soda with her meal but drank water during the rest of the day. Another participant described the times her family drinks soda when she said:

“We buy them (soda), yes, we drink them, but with certain foods… For instance, with pizza, hamburgers, when you make (these foods) at home… you drink a soda.”

Various participants emphasized that when high sugar foods, particularly SSB, were available in their home environments they were likely to consume those frequently. A number of participants reported that they regularly maintained SSB in their homes and consumed them often because they were easily available. Several others spoke about using avoidance strategy to limit SSB by not keeping them in the home, but often struggled in limiting their consumption when other family members would purchase them. One participant recalled telling her husband “*if you don’t want me to drink soda, do not buy soda, not even for the visitors*.”

All the participants emphasized the impact of holidays on sugars consumption in their diet. The women in our study spoke of “holidays” in terms of holiday seasons (e.g., Christmas season) as well as other celebrations such as birthday parties; they described these as events that centered on social gatherings with friends and family, typically involving presentation of traditional Mexican foods. For example, participants reported that some of the traditional foods and beverages commonly prepared and served during holidays, like *Piloncillo*, contain large amounts of sugars, contributing to their increased sugars consumption. They also spoke about increasing their consumption of high sugar drinks like punches, *atole* [a hot drink that is made with corn starch] and hot chocolate during the holidays.

Many of the women explained that their increased sugars intake during the holidays resulted from attending many different social gatherings where they are expected to eat at each stop, otherwise they would be perceived as being rude. Furthermore, holidays are the time when family members are brought together to prepare and enjoy traditional holiday foods. This is exemplified in one participant’s statement:

“(During) Christmas season when it is cold, we make cookies to have at home… The girls are on vacation already, we are all at home and it is when they get ready to bake… we are all together and doing something.”

One participant summarized the increase in calorie-dense and high-sugar foods associated with holidays in the following statement: *“well, yes during the holidays, we eat everything, desserts, tamales [a corn-based dough steamed/boiled in corn husks with sweet or savory filling]*, *pozole* [a flavorful soup made with corn], *everything that is there. Desserts, soda, yes, but the fruit (laughs) is not included.”*

## DISCUSSION

Our work demonstrates several important considerations regarding sugars consumption in low-income Mexican-American women: 1) the critical differences between food procurement in Mexico and in the U.S., 2) family values in food procurement, food preparation, and food presentation, 3) the significance of family relationships and gatherings in food preparation, and 4) the reliance on traditions in food presentation.

### Critical Differences between Food Procurement in Mexico and in the U.S.

Consistent with literature on nutritional changes post-migration (Guarnaccia et al., 2012; Himmelgreen et al., 2007; Jay et al., 2014), participants in our study spoke about purchasing more SSB and high sugar foods in the U.S. than they did in their native country. This is similar to a participant’s statement in Himmelgreen et al.’s (2007) study that SSB are convenient and that “*we don’t have the same fruits or the time to (prepare natural juices)*.” The increase in the consumption of sugars and highly processed foods may be related to the prestige or high status given to certain “American” foods. For some Latinos, the significance of these foods is linked to having a higher economic status, as many of the foods that were not available in their countries (or were reserved for special occasions/celebrations) are affordable and easily available in the U.S. Consumption of such foods gives them a sense of belonging/fitting into the American culture (Guarnaccia et al., 2012; Himmelgreen et al., 2007; Lindberg & Stevens, 2011), thereby increasing the desirability of such foods (Himmelgreen et al., 2007). The change in purchasing behavior is also determined by limited access to farming or fresh produce compared to rural Mexico, and higher access to convenient stores in the U.S. (Ayala et al., 2008; Batis et al, 2011).

Participants in our study discussed that selection of foods purchased and prepared in the U.S. was driven by cost; that the low cost of junk foods and SSB in the U.S influenced their decision to purchase and consume such foods. This finding was contrary to Smith et al.’s study (2016), which found that cost of food was not a decisive factor in food purchasing and that healthy foods were highly accessible in participants’ communities. The authors of this study report that their study sample had a relatively higher socioeconomic status than Mexican-American population in the U.S. (Gonzalez-Barrera & Lopez, 2013; Smith et al., 2016). This discrepancy reinforces the importance of acknowledging the diversity of the Latino population in the U.S., and considering socioeconomic factors in the development of future interventions.

Our study results were consistent with literature on the relationship between the fast pace of life in the U.S., decreased time for food preparation (Gray, Cossman, Dodson, & Byrd, 2005; Himmelgreen et al., 2007), and increased consumption of processed foods and SSB among Latino immigrants in the U.S. (Himmelgreen et al., 2007). One of the primary distinctions in terms of food procurement was found in the amount of time devoted to procuring food in Mexico as opposed to the amount of time devoted to this activity in the U.S. Participants reported that the fast pace of life in the U.S. and long work schedules resulted in a lack of time for purchasing and preparing foods as was done in Mexico. Our participants reported buying more calorie-dense items and convenient foods due to time constraints, juggling jobs and children, which is a frequent notation in the literature: “*it is simply easier to grab something on the way home*” (Guarnaccia et al., 2012).

### Family Values in Food Purchases

Similar to findings by Jay et al. (2014), participants in our study described their family members as being influential, i.e., being either supportive or unsupportive, for making healthy lifestyle choices. In particular, as Ayala et al. (2008) showed, children were a significant influence of dietary choices and shopping selections. Our Mexican-American mothers stated that, despite having some knowledge about what constitutes healthy nutrition, they often acquiesced to children’s requests for sweets, SSB and fast foods. Purchasing of these foods may be influenced by Mexican-American mothers’ desires to please their family members and help them fit into American lifestyles. Similarly, Heuman et al. (2013) found that sugary foods were often used as rewards or treats by parents, and a way to communicate affection for their children. In our focus groups, we found that males/spouses also purchased and prepared foods with the goal of pleasing others.

#### Significance of Family Relationships and Gatherings in Food Preparation

In Mexico, food rituals, that is rituals at which a number of people are fed a meal where food is served in an artful or attractive manner, and meals can last two or three hours, are quite different from the simple food rituals commonly seen in the U.S, which often involve eating or snacking alone. In the 2011–2013 American Time Use Survey, Sliwa at al (2015) found that although full-time working Latinas spent 38 fewer minutes per day in food preparation compared to non-working Latinas, they did not sacrifice their time spent in family dinners, supporting the importance of family meals in this culture. While it is recognized that mealtime structure plays an important role in the eating patterns that children develop and are likely to maintain over their lifetime (Patrick & Nicklas, 2005), the structure also influences the amount that is consumed by adults related to the amount and type of social interaction at the meal.

The women in our focus groups spoke of food preparation as a process involving various family members; this was consistent with a study with Mexican-American women by Lindberg and Stevens (2011) in which participants described food preparation being a family affair involving multiple family and extended family members, or how they learned preparing traditional recipes from their mothers, older sisters or mothers-in-law. Participants in our study described similar patterns of food preparation during holidays. They spoke of changes in their family’s diet when their husbands, mothers or in-laws were in charge of food preparation, often resulting in the woman’s lack of control over how to feed her family (e.g., mother-in-law adds high amounts of sugar to everything). A similar finding was reported by participants in Smith et al. (2016) who spoke of the tension that resulted from family members of younger generations preparing traditional foods in a healthier manner. For example, one participant in their study spoke about the difficulty in trying to maintain a healthy diet despite her mother continued use of lard in cooking (Smith et al., 2016). Further added to this conflict is the challenge of children not wanting to eat traditional Mexican foods and only wanting to eat “American” foods such hamburgers, hot dogs, and soda. This finding is consistent with a study by Pyatak et al. (2014), which reported conflicts in Latino families resulting from disagreements and differing views between parents on how to feed their children. Such findings reinforce the importance of involving family members, recognizing the importance of females, and the role that they play in shaping dietary behavior in the household (e.g. mother-in-law), understanding family dynamics, and teaching collaboration/negotiation strategies in food preparation in future dietary and nutrition interventions with Mexican-American women.

### Reliance on Traditions in Food Preparation and Presentation

Participants in our study, as well as Smith et al.’s (2016), spoke about attending frequent social events such as weddings and *quinceañeras* (traditional celebration of a girl’s 15^th^ birthday) that involved large spreads of homemade dishes, and high-sugar drinks, like punches, cocktails, *atole* and hot chocolate. Similarly to what was reported by Lindberg and Stevens (2011), the women in our study confirmed that food plays a central role in the Mexican-American culture, not only in day-to-day life but during these holidays and celebrations. Participants spoke of their struggles with maintaining a healthy diet during food-centered celebrations. During the holidays they would often go to several social gathering in a day and were expected to eat at each one. This finding was similar to Lindberg and Stevens’s study (2011) in which Mexican-American women reported social and family pressures to take part in food-centered celebrations and avoid offending others by not eating at such gatherings.

Interestingly, although the women expressed strong ties to their culture and traditional foods, they also spoke of celebrating typically U.S.-based holidays such as Thanksgiving. Given the extensive length of the holiday seasons (e.g., November-January), and frequency of celebrations such as birthdays/*quinceañeras*, these celebration periods provide an ideal opportunity for interventions to promote healthy nutrition and sugar reduction strategies.

In our study, participants provided examples of behavioral patterns and routines relating to food consumption that rapidly became established. For example, one participant expressed that her 10 year-old made cakes and cookies and served them in an attractive manner. The participant stated *“we say how good they are and she makes more*.” With ongoing reinforcement from others, her daughter has established a routine of baking sweets several times weekly. While such behaviors may be unintentionally promoted through reinforcement, they can lead to the establishment of long-term unhealthy dietary habits; however, similar behavioral strategies could also be used to develop healthy dietary patterns and routines (e.g., teaching children how to make tortillas). Participants in our study demonstrated low nutrition knowledge and misinformation about nutritional content of foods they purchased and prepared, and stated that they often did not have the time to read labels in supermarket or did not understand what the labels meant. These findings are similar to the report by Lindberg and Stevens (2011) that “*the only number you read is the price*.” Despite the lack of knowledge, our participants expressed eagerness to learn about the nutritional value of foods, how to read food labels and for attending cooking demonstrations where they could share traditional Mexican recipes and learn ways to prepare traditional dishes in healthier ways.

Our small sample of low-income Mexican-American women of childbearing age who have been in the U.S. for more than 8 years limits the generalizability of findings to a broader population of Latina adults, especially to those who have recently migrated, are older or of higher socio-economic status. All our women reported annual household income of less than $51,200 for a family of four, which is the low-income limit for the Phoenix Metropolitan Area for year 2015 (Office of Policy Development and Research, 2015), and were of similar age. Despite the small size, our study provides useful information on consumption of sugars, an important modifiable risk factor, in this largest ethnic subgroup in the U.S, and contributes to the paucity in literature on this topic. Out of our 13 participants, 12 were responsible for food purchasing and all were responsible for meal preparation in the household. Hispanic women are traditionally responsible for shopping and preparing food, and largely influence the home food environment (Evans et al, 2011). Gaining understanding of the sugars consumption behavior in women of childbearing age may be crucial for early childhood obesity prevention in this population, given the evidence that consumption of sweets and SSBs among low-income Hispanic women is a strong determinant of infant weight (Watt et al, 2013). We identified culturally-relevant strategies that are ready to be leveraged in behavioral interventions to reduce sugar consumption in low-income Mexican-American mothers. Addressing such cultural and contextual factors of dietary behavior is essential for reducing sugars intake and related health conditions in this underserved population.

## CONCLUSION

Future interventions must acknowledge the value of social ties and the integral role that each family/extended family member plays in influencing diet, as well as the importance of maintaining traditional foods and cultural celebrations. Based on our findings, the following strategies can be used in future interventions to promote healthy nutrition and reduce sugars intake in Mexican-American women. First, maintain food traditions and rituals (purchase fresh fruit as they used to in native Mexico; drink water instead of SSB) while incorporating healthier modifications (e.g., adding herbs to *aguas frescas* for flavor instead of sugar, mixing fruit with vegetable juice, tweaking the traditional recipe to include less sugar), or establish new healthier traditions. Second, teach strategies for time- and cost-efficient food procurement, preparation, and presentation (e.g., how to choose affordable low-sugar foods; read and interpret sugar content on food labels; determine portion sizes; choose seasonal or frozen fruits; for those enrolled in the Supplemental Nutrition Assistance Program, use your benefits to purchase fresh fruits and vegetables at farmers markets). Third, reinforce the importance of Latina’s role modeling healthy dietary behaviors to their children, teaching by example (e.g., abstaining from consumption of SSB and processed food). Fourth, teach strategies for negotiating healthy dietary practices with family members (e.g., what to do when in-laws prepare high sugar food; husbands bring home SSB and fast foods). Last, adopt a family-based approach where all family members share the same goals and challenges related to nutrition consumption and sugars intake.

## Figures and Tables

**Table 1.** Sample Questions from Focus Group Script

Topic	Question/Information Elicited
**Food Procurement**	Tell me about your grocery shopping. Who does the household shopping? How often do you buy sweets, sugary foods and soda, and which ones do you buy most often? Who influences these purchases? Tell me about how your consumption of sweets and sugary drinks changes when someone else buys food? (Does their sugar intake differ when other members buy the food?) How has your shopping changed since coming to the U.S.?
**Food Preparation**	Tell me about how you learned to prepare, cook, and bake cakes/cookies/desserts? How often do you make them? Tell me about how you learned to prepare the traditional sweet drinks *(horchatas, atole*, fruit punches, etc.) and cocktails? How often do you make them? Tell me about how your consumption of sweets and sugary drinks changes when someone else is cooking? Prompt: Does sugar intake differ when other members prepare the food or buy the food?
**Food Presentation**	Tell me about how eating sweets/desserts and drinking sugary drinks have changed since you were a kid to now when you’re having kids. Prompt: How has their sugar consumption changed throughout their life? Where did they learn their sugar consumption behaviors? Tell me about how Holidays or Birthdays change the amount and type of sweets/desserts and sugary drinks you consume.
**Recommendations/Strategies**	Tell me about what would help you eat less sweets and sugary foods and drinks (e.g., social support systems, social media) Tell me about what motivates you to reduce the amount of sweets and sugary foods and drinks you and your family consume.

**Table 2.** Demographic Characteristics of Study Participant (n = 13)

Characteristic	n (%)
**Mexican-American female**	13 (100)
**Age, years** (mean, SD)	35.3 (4.0)
**BMI, kg/m**^**2**^ (mean, SD)	29.7 (3.1)
**Preferred Language**	
Spanish	11 (84.6)
English	1 (7.7)
“Spanglish”	1 (7.7)
**Marital Status**	
Married	8 (61.5)
Living with a partner	1 (7.7)
Single, never married	3 (23.1)
Divorced	1 (7.7)
**Annual Household Income**	
Less than $20,000	5 (38.4)
$20,000-$29,999	4 (30.8)
$30,000-$49,999	4 (30.8)
**Employment Status**	
Full-time (35–45 hrs/week)	4 (30.4)
Part-time (less than 15 hrs/week)	3 (23.1)
Not employed	3 (23.1)
**Education**	
Less than high school	5 (38.5)
High school graduate	3 (23.1)
Attended some college	2 (15.4)
Bachelor’s degree	3 (23.1)
**Household size**	
2–3 individuals	2 (15.4)
4–5 individuals	5 (38.2)
6 or more	6 (46.2)
**Who does the majority of household food shopping?**	
Participant	12 (92.3)
Other (in-law)	1 (7.7)
**Who does the majority of household food preparation?**	
Participant	13 (100)
**Who do you seek dietary advice from?**	
Medical Doctor	4 (30.8)
Nutritionist	5 (38.4)
Other	2 (15.4)
No one	2 (15.4)

## APPENDIX

### Focus Group Study Script

Questions we hope to answer in the discussion: (for interview leader only- a lay description is below)

1. What socio-cultural factors influence sugars intake?
2. What are the barriers to consuming a diet low in sugars?
3. What are the facilitators to consuming a diet low in sugars?

Facilitator Key:

**Table:**

Questions/Statements to read directly to the group
*Facilitator instructions/notes (do not read these to the group)*
Please ask participants to expand their responses and ask follow up questions, as needed, that make sense for the conversation in order to get the answer to the “things we hope to gain” for each question.

Focus Group Questions/Script:

**Table:**

*Thank you for facilitating this focus group. Throughout the discussion, you may utilize key phrases like:* Tell me more about… (to gather more information) Does everyone agree with that? (to provoke agreement or disagreement) What I hear you saying is… (to summarize) Can you talk a little more about… (to gather more information) Are there any other thoughts? (to elicit others to speak) Just to clarify… (to clarify something said)
*Set the stage to help participants feel comfortable, safe and confident*. **Welcome to our discussion. Thank you for coming and participating today. Please introduce yourself to the group.**
*Explain consent form and their participation protection. Participants should be informed of the purpose of the study, including potential risks and benefits of study participation and will be given the opportunity to participate or refuse. They are free to exit the study at any time. Participants will be assured of confidentiality in their responses on the baseline questionnaire and during the focus group session. Before completing the baseline questionnaire, participants should sign an informed consent, available in Spanish and English. Women who are invited to participate are: (i) participants or promotoras in the Madres study or friends and family of those individuals; (ii) aged 27–40 years; and (iii) having a BMI between 25 and 40 kg/m*^*2*^*. Women are not invited of they: (i) take medications or are diagnosed with a syndrome or disease that could influence diet; or (ii) have been enrolled in any weight management program over the past 3 months*.
*Review purpose of their invitation: We want to develop a program for young Latinas to help make decisions about their diet that will help them reduce their risk for cardiovascular disease and diabetes. To do that, we are asking for your help to help us learn about Latina cultural and traditional management of your family’s eating habits. We also want to learn if things like shopping or preparing food have changed for you as you moved from childhood to young adulthood and mothers*.
**We are most interested in eating sugars: sugars are added in foods and drinks such as *horchatas*, atole, fruit punches and regular soda, sugared teas, high-sugar milk products (ice cream, sweetened yogurt, sweetened milk), sweets and snack foods, and deserts (e.g., *pan dulce, cajeta/dulce de leche, flan)*.**
**This interview/focus group will be a conversation among the women who are here today. The group leader will ask questions to stimulate discussion about dietary practices, conducted in Spanish and English by a bilingual bicultural moderator and an assistant moderator, and will last approximately one hour and a half.**
**These questions will ask you to think and talk about your mothers cooking and food habits, family influences, your influence on your family’s eating.**
**We will be recording the conversation and taking notes. If you use your name or a member of the group’s name, please do not use last names.**
**Any questions before we get started? Okay, let’s get started!**
**1) Tell me about your grocery shopping. Who does the household shopping?** **How often do you buy sweets, sugary foods and sweet drinks and which ones do you buy most often? How has your shopping changed since coming to the U.S.?** Information we hope to gain: Do they recognize the foods that are high in sugars and what foods are they? Who influences these purchases? How often do they buy sweets or drink sweet beverages
**2) Tell me about how you learned to prepare, cook, and bake the sweet and sugary foods that you like to eat and how often do you make them.** *Information we hope to gain:* Who taught them to use sugars Do they use recipes and if so do they put a lot of sugar or try to cut or use replacements Do they usually buy them or prepare them
**3) Tell me about how you learned to prepare traditional sweet drinks *(horchatas, atole*, fruit punches, etc.) and alcoholic drinks *(cocktails)* and how often do you make them?** *Information we hope to gain:* Who taught them to add sugars How often do they consume alcohol How often do they consume sweet beverages Is it the whole family that consumes at that rate or just them How much sugar do they add to drinks
**4) Tell me about how your eating of sweets and sugary drinks has changed since coming to the U.S./since you were a kid to now when you’re having kids.** *Information we hope to gain:* Has their sugar consumption changed throughout their life Where did they learn their sugar consumption behaviors
**5) Tell me about when someone else is cooking or buying the food. How does that change the amount of sweets and sugary drinks you eat?** *Information we hope to gain:* Does their sugar intake differ when other members prepare the food or buy the food
**6) Tell me about how the holidays or Birthdays and any change in how much or type of sweets and sugary drinks you eat compared to normal.** *Information we hope to gain:* Do they consume more sugars during the holidays Are there any specific types of foods/beverages (including alcoholic) that they drink only during the holidays? Do they refrain from eating more or do they not care? When they go to different gatherings is it mostly sweets and alcoholic beverages?
**7) Tell me about what would help you make changes in how sweets & sugary foods and drinks you eat.** *Information we hope to gain:* How can their social support systems help them meet their sugar goals Would speaking to someone be helpful How often and how, would they want to be contacted via social media
**8) Tell me about what motivates you to want to change the amount of sweets and sugary foods and drinks you and your family eat.** What motivates them?
**9) Tell me about anything else that plays a role in how much sweets and sugary foods and drinks you eat.** Anything we may have missed.
